# Data for proteomic analysis of murine cardiomyocytic HL-1 cells treated with siRNA against tissue factor

**DOI:** 10.1016/j.dib.2015.02.005

**Published:** 2015-02-25

**Authors:** Maura Brioschi, Sabrina Lento, Simona Barcella, Md. Talat Nasim, Stefania Ghilardi, Silvia Stella Barbieri, Elena Tremoli, Cristina Banfi

**Affiliations:** a Centro Cardiologico Monzino IRCCS, Milano, Italy; b National Institute for Health Research Comprehensive Biomedical Research Centre, King׳s College London, UK; c Department of Medical and Molecular Genetics, King׳s College London, UK; d Bradford School of Pharmacy, School of Life Sciences, University of Bradford, UK; e Dipartimento di Scienze Farmacologiche e Biomolecolari, Università degli Studi di Milano, Milano, Italy

## Abstract

This data article is related to the research article entitled Proteomics of Tissue Factor silencing in cardiomyocytic cells reveals a new role for this coagulation factor in splicing machinery control by Lento et al. [1].

Tissue Factor (TF) is a key player in the coagulation cascade, but it has additional functions ranging from angiogenesis, tumour invasion and, in the heart, the maintenance of the integrity of cardiac cells. This article reports the nano-LC–MS^E^ analysis of the cardiomyocytic HL-1 cell line proteome and describes the results obtained from a Gene Ontology analysis of those proteins affected by TF-gene silencing.

**Specifications table**

**Table:** 

Subject area	*Biology*
More specific subject area	*Cellular proteomics*
Type of data	*Excel files*
How data was acquired	*Experiments were performed on the hybrid quadrupole-time of flight mass spectrometer SYNAPT-G1 (Waters corporation, Milford, MA, USA) coupled to the nanoAQUITY UPLC system (Waters corporation, Milford, MA, USA)*
Data format	*Processed data*
Experimental factors	Cardiomyocytic HL-1 cells were treated with TF siRNA or a non-silencing oligonucleotide sequence
Experimental features	*Cell lysates were digested with trypsin and analysed by nano-LC–MS^E^ and processed with PLGS 2.3 (Waters corporation, Milford, MA, USA)*
Data source location	*Milan, Italy*
Data accessibility	*Data are provided with this article and are related to**[1]*

## Value of the data

• 370 proteins were identified in the cardiomyocytic HL-1 cell line proteome.
• The data are valuable for the understanding of the protein composition if cardiomyocytes and could be reused by other scientists investigating these cells under various conditions.
• Computational analysis of differentially expressed proteins following TF gene silencing revealed a novel role of this coagulation factor in the regulation of splicing machinery.

## 1 Data, experimental design, materials and methods

Cardiomyocitic HL-1 cells were treated with siRNA against TF which resulted a 83.7±5.6% reduction of TF mRNA levels in comparison with cells treated with a non-silencing oligonucleotide sequence. Cell lysates were digested with trypsin and their proteome were compared by a label free nano-LC–MS^E^ analysis which allowed both a qualitative and quantitative analysis of 370 proteins (Supplementary Table 1). Differentially expressed proteins were further investigated with computational analysis for the identification of over-represented GO categories (Supplementary Table 2).

### 1.1 Cell cultures, RNA interference and cell transfection

The HL-1 cardiomyocytes, gift of Prof. Claycomb (LSU Health Sciences Center, New Orleans, LA, USA), were cultured according to Prof. Claycomb׳s instructions [2]. Gene silencing was performed using small interfering RNAs (siRNA) against TF or a nonsilencing oligonucleotide sequence provided by Qiagen Inc. following the manufacturer׳s instructions as described in [1].

### 1.2 Label-free LC–MS^E^ analysis

The cell lysates, dissolved in 25 mmol/L NH4HCO3 containing 0.1% RapiGest (Waters Corporation, Milford, MA, USA) were digested as previously described [3]. The tryptic peptides were analysed by means of a nanoACQUITY system coupled to a SYNAPT-MS, a hybrid Q-TOF mass spectrometer (Waters Corporation, Milford, MA, USA), for the LC–MS^E^ analysis as previously described [4].

ProteinLynx GlobalSERVER (PLGS) v 2.3 (Waters Corporation, Milford, MA, USA)was used foriIon detection, data clustering, and database search of the data-independent LC–MS^E^ data as previously explained in detail [1,5,6]. The entire data set of differentially expressed proteins was further filtered by considering only the identifications from data with identified peptides that replicated at least two out of three technical instrument replicates and in two out of three biological replicates [1].

### 1.3 Computational analysis

The list of proteins down-regulated by TF-silencing were further analysed with the BiNGO plugin (v 2.3) in the Cytoscape (v 2.7) software platform in order to make gene ontology (GO) assignments and identify over-represented GO categories for cell component and biological, as previously described [3]. Statistical analysis was obtained using the hypergeometric analysis followed by Benjamini and Hochberg׳s false discovery rate correction (*p*<0.001) [7].
